# A Comprehensive Perspective on the Biological Effects of Intermittent Fasting and Periodic Short-Term Fasting: A Promising Strategy for Optimizing Metabolic Health

**DOI:** 10.3390/nu17132061

**Published:** 2025-06-20

**Authors:** Barbara Ciastek, Karolina Kapłon, Przemysław Domaszewski

**Affiliations:** Institute of Health Sciences, University of Opole, Katowicka 68, 45-060 Opole, Poland; karolina.kaplon@uni.opole.pl (K.K.); przemyslaw.domaszewski@uni.opole.pl (P.D.)

**Keywords:** intermittent fasting, periodic short-term fasting, metabolic changes, signaling pathways

## Abstract

It is well established that different fasting strategies offer a range of benefits and may even serve as potential therapeutic approaches for metabolic diseases. The biological effects of intermittent fasting (IF) are multidimensional, involving the induction of metabolic switching from glucose to fatty acid and ketone utilization, thereby enhancing fat metabolism and improving glucose tolerance and insulin sensitivity. In addition, IF modulates the growth hormone/insulin-like growth factor 1 (GH/IGF-1) axis by lowering IGF-1 levels, a change associated with enhanced cellular protection, reduced tumorigenesis, and delayed aging. Moreover, IF modulates key signaling pathways, including mitogen-activated protein kinases, Notch, and nuclear factor kappa B, which collectively contribute to reduced oxidative stress, attenuated inflammation, and hepatoprotection. Although fasting may present certain challenges, it is essential to be adequately informed about its potential benefits and appropriate preparatory strategies before undertaking various fasting protocols. This review summarizes the current knowledge on various IF protocols and periodic short-term fasting (PSTF) lasting more than 24 h and up to 72 h, highlighting the signaling pathways through which these interventions affect metabolic processes. Additionally, it aims to provide a practical guide for the safe preparation for PSTF lasting more than 24 h and up to 72 h.

## 1. Introduction

Hypertension (HTN), type 2 diabetes mellitus (T2DM), hyperlipidemia (HLD), obesity, and metabolic dysfunction-associated steatotic liver disease (MASLD) are all classified as metabolic diseases and rely on intricate metabolic interactions [1,2]. According to data from the Global Burden of Disease, Injuries, and Risk Factors Study (GBD) 1990–2021, the global burden of metabolic diseases in 2021 included 226 million cases of HTN, 129 million cases of obesity, 88 million cases of HLD, 75 million cases of T2DM, and 3.67 million cases of MASLD [2]. Collectively, these five metabolic conditions affected more than 521 million individuals globally, corresponding to approximately one in every fifteen people worldwide, or roughly 6.6% of the global population, who were affected by at least one of these disorders. Over the past three decades, the global burden of these conditions has increased markedly, with prevalence rates rising approximately 1.6- to 3-fold [2].

Obesity is a major driver of the rising global burden of metabolic diseases. This alarming trajectory underscores the urgent need for effective public health interventions [3,4]. Notably, estimates of the global burden of obesity vary considerably depending on the source and methodology used. As previously mentioned, the GBD reported approximately 129 million obesity-related cases worldwide in 2021, based on metrics linked to disease burden and disability. In contrast, the World Health Organization (WHO) estimated that in 2022, nearly 890 million adults globally were classified as obese, based on population-level surveillance using body mass index criteria (BMI ≥ 30) [2,5]. The effective treatment and prevention of metabolic diseases pose significant global health challenges due to their latent onset, progressive nature, and typically slow development [6]. Metabolic diseases are strongly associated with lifestyle-related factors, many of which are preventable, including low-nutrient-density diets, tobacco use, alcohol consumption, and physical inactivity [7]. The effective treatment of metabolic diseases requires a comprehensive approach that integrates lifestyle modifications, pharmacological interventions, and, in some cases, surgical procedures. However, despite the implementation of various preventive and therapeutic strategies, the global burden of these diseases continues to grow, highlighting the urgent need for more effective and innovative management approaches [8].

In recent years, there has been a growing trend among patients to explore alternative, non-pharmacological, and dietary strategies to improve health outcomes, often in conjunction with or as an alternative to conventional medical treatments [1]. Could intermittent fasting (IF) gain wider recognition and emerge as a sustainable long-term strategy for managing metabolic disorders? Might revisiting ancient practices offer modern solutions? Fasting, a practice deeply embedded in ancient cultural traditions, is experiencing a resurgence as a potentially effective therapeutic approach for both the prevention and management of metabolic diseases. Otto Folin, in the early 20th century, advocated the therapeutic use of fasting in the treatment of obesity [9].

Fasting is defined as the voluntary abstinence from solid food and caloric beverages (but not water) for a specific period. It has traditionally been practiced for religious, spiritual, ethical, or cultural reasons, but is increasingly adopted for health-related purposes [10,11]. The IF schedule can be categorized into alternate-day fasting (ADF) or time-restricted eating (TRE) patterns, with or without strict calorie restriction, allowing individuals to choose a fasting schedule that best fits their lifestyle and goals. TRE includes some of the most popular methods including the 16/8 approach (fasting for 16 h and eating within an 8 h window) and the 20/4 method (fasting for 20 h with a 4 h eating window) [10,11,12]. Apart from the aforementioned fasting regimens, periodic short-term fasting (PSTF) represents an alternative approach, typically involving water-only fasting periods lasting more than 24 h and up to 72 h [13,14,15,16]. The duration of fasting may extend to several dozen days; however, fasting for more than 72 h should not be undertaken without medical supervision due to the risk of malnutrition, excessive drops in blood pressure, or severe hypoglycemia [14].

**Figure 1:**
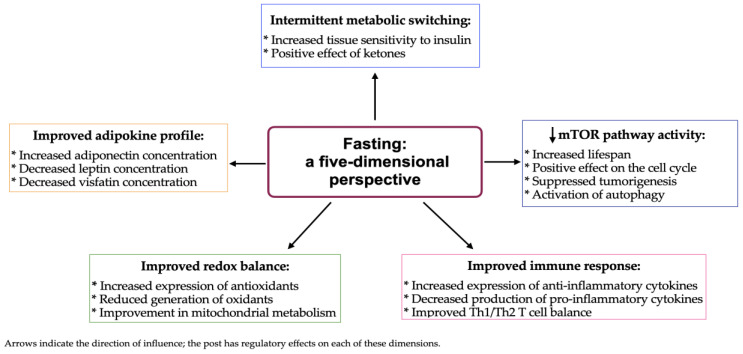
Mechanisms of fasting and their influence on body metabolism.

As depicted in Figure 1, the biological effects of fasting are multidimensional, initiating a metabolic transition in which glucose is supplanted by fatty acids and ketone bodies as the principal energy substrates, a process referred to as intermittent metabolic switching. This adaptive mechanism promotes lipid oxidation, reduces adipose tissue accumulation, and contributes to favorable alterations in the lipid profile [13,17,18]. Thus, it may lead to dynamic cellular alterations that manifest as measurable clinical outcomes, i.e., improved glucose tolerance and insulin sensitivity via reductions in insulin and leptin levels, enhanced adiponectin secretion, and improved redox balance [12,19]. Periodic short-term fasting (PSTF), lasting more than 24 h and up to 72 h, has been associated with pronounced metabolic benefits, including the upregulation of autophagy, attenuation of aging mechanisms, and enhanced modulation of critical cellular signaling pathways, which collectively facilitate the restoration of cellular homeostasis [13,14,15]. Interestingly, both cellular and systemic biological responses are particularly evident in individuals adhering to a daily IF routine.

Although the benefits of various fasting methods are well recognized, it is important to acknowledge potential complications, such as intensified hunger, headaches, electrolyte imbalances, dehydration, dizziness, and so on, that often lead individuals to discontinue fasting. A thoughtful and personalized approach to fasting is therefore essential, and long-term adherence may depend on adopting IF as a lifestyle rather than a temporary strategy for weight loss and caloric restriction. This review explores the current body of knowledge, including both the benefits and limitations, regarding the effects of various IF protocols and PSTF lasting more than 24 h and up to 72 h on metabolic and signaling pathways. Additionally, it aims to provide a practical guide for the safe preparation for PSTF within this timeframe.

## 2. Impact of Various Fasting Regimens on Metabolic and Cellular Signaling Pathways

### 2.1. Overview of Different Intermittent Fasting Patterns

IF encompasses a variety of dietary regimens, including ADF, TRE, one meal a day (OMAD), and the 5:2 diet. ADF typically involves alternating 24 h periods of fasting with days of unrestricted eating several times per week (Table 1) [20,21]. In contrast, TRE confines daily food intake to a defined time window, usually between 4 and 10 h, without necessarily reducing total caloric intake, followed by a prolonged fasting phase [21,22,23]. Recent studies have demonstrated that TRE can yield notable metabolic benefits, particularly in older adults who are overweight or obese. Research indicates that TRE facilitates reductions in body weight, visceral fat, and waist circumference without negatively affecting skeletal muscle mass, which is vital for maintaining physical function in aging populations [24,25]. Table 1 provides an overview of the most widely practiced IF approaches, including their key features, strengths, and challenges [16,21,22,23,26,27].

### 2.2. Effect of Various TRE Schemes on Metabolic and Signaling Pathways

The mechanisms underpinning TRE are closely linked to circadian rhythms, improving metabolic efficiency and enhancing, i.e., insulin sensitivity. Additionally, TRE has been found to influence oxidative stress pathways and the gut microbiome, further contributing to its wide-ranging health benefits. Notably, adherence rates to TRE protocols have been exceptionally high, often exceeding 95%, suggesting that it is a sustainable dietary approach over the long term [28,29]. Given its physiological benefits and ease of adherence, TRE presents a highly promising nutritional strategy to reduce obesity, improve overall health, counteract age-related metabolic decline and promote longevity [29]. Among the various TRE protocols, the 16/8 model is the most extensively studied and widely adopted. In contrast, considerably fewer studies have examined the effects of the 18/6 regimen, in which the eating window is restricted to six hours. Research on the 20/4 fasting pattern remains particularly limited, with only a small number of studies assessing the impact of this TRE strategy on human health. The divergent results may also be related to the characteristics of the patient groups analyzed, such as whether they were healthy, obese, overweight, or diabetic. Notably, in healthy individuals, these effects tended to be more pronounced and longer-lasting.

The benefits of IF can be categorized into short- and long-term effects. Short-term outcomes include weight loss, improved DNA integrity, and reduced fat mass, whereas long-term benefits involve a markedly lower risk of metabolic disease development. Additionally, beyond its metabolic advantages, IF shows therapeutic potential in oncology by mitigating chemotherapy side effects and inhibiting tumor growth [1]. Data suggest that this effect is largely mediated by suppression of the protein kinase B (AKT)/mammalian target of rapamycin (mTOR) signaling pathway, driven by reduced circulating levels of insulin and insulin-like growth factor 1 (IGF-1), along with increased concentrations of IGF-1 binding proteins 1 and 2 (IGFBP-1 and IGFBP-2) [30,31]. IF may further exert protective effects against neoplastic transformation by attenuating systemic inflammation, as indicated by decreased levels of interleukin 1 (IL-1), interleukin 6 (IL-6), and tumor necrosis factor α (TNF-α) [21,32], by improving the adipokine profile (through decreased leptin and increased adiponectin levels) [32], and, importantly, by lowering sex hormone levels [32,33] and their bioavailability (Figure 1) [31,34].

Table 2 provides a comprehensive overview of studies investigating the biological effects of various TRE protocols in human participants. It includes key details such as positive and negative metabolic outcomes, study populations, study designs, and specific fasting and eating windows. In summary, based on the data presented in Table 1, we conclude that further studies involving larger populations and extended follow-up periods are warranted to determine and confirm the long-term benefits of TRE, irrespective of the specific protocol implemented.

### 2.3. Effect of Various PSTF Schemes on Metabolic and Signaling Pathways

As highlighted earlier, non-communicable diseases like obesity and diabetes constitute a growing global health crisis. This represents a striking paradox: within just one century, global societies have shifted toward excess, characterized by widespread overweight and obesity, and driven by overconsumption, where the prevalence of obesity now surpasses that of starvation, especially in the Western world. Despite the abundance of food, malnutrition persists due to the widespread availability of highly processed, visually appealing, carbohydrate-rich foods that lack essential nutrients. According to the WHO, in 2023, approximately 733 million people, about 1 in every 11 globally, faced famine. In stark contrast, in 2022, 890 million people, roughly 1 in every eight were classified as obese, while a staggering 2.5 billion adults aged 18 years and older were considered overweight [5,53]. Apart from the burden of obesity, one of the most critical underlying issues is insulin resistance and the resulting hyperinsulinemia. According to data from the National Health and Nutrition Examination Survey 2021, 40% of U.S. adults aged 18 to 44 are affected by insulin resistance (IR), as indicated by the homeostasis model assessment of insulin resistance (HOMA-IR) [54]. Meanwhile, global rates of insulin resistance range from 15.5% to 46.5% [55]. IR contributes to the activation of a vicious cycle involving excessive appetite, subclinical inflammation, shifts toward oxidative stress, and excessive adipose tissue accumulation. Interestingly, IR can be reversed or significantly reduced through a simple, low-cost intervention involving 48 to 72 h of PSTF. Additionally, the beneficial effects of PSTF may be further enhanced when IF is incorporated into the daily routine.

Water-only fasting refers to the complete abstinence from food while allowing unlimited water intake. It is commonly practiced for health, therapeutic, or spiritual purposes. The duration of such fasts can range from short periods (24–48 h) to extended fasts lasting several days or even weeks. Longer fasts should be undertaken only under medical supervision [56,57]. Even when PSTF is performed within a safe time frame, lasting more than 24 h and up to 72 h, it remains insufficiently researched, and there is a limited number of studies available on this specifically (Table 3). Additionally, fasting often carries culturally and socially ambiguous connotations, frequently associated with poverty or involuntary food deprivation. Overcoming the longstanding perception that fasting is inherently harmful continues to pose a significant social challenge.

Various fasting patterns have been associated with significant reductions in body weight, blood glucose, and insulin levels, thereby improving insulin sensitivity. Additional reported benefits include enhancements in lipid profiles, lowered blood pressure, and other favorable metabolic adaptations (Table 3) [15,51,58,59,60,61,62,63,64,65,66,67,68,69,70,71,72,73]. Despite the numerous benefits of different fasting protocols (48–72 h), individuals may experience or report an exacerbation of symptoms such as reduced well-being, feelings of guilt, impaired decision-making, hunger, decreased libido, and dizziness [59]. Wilhelmi de Toledo et al. reported that the most frequently observed mild symptoms of fasting may include sleep disturbances, dry mouth, back pain, muscle pain, abdominal bloating, bad breath, sensitivity to cold, and blurred vision, among others [57]. In a study by Ding et al., the Beck Depression Inventory-2 (BDI-2) score significantly increased after 72 h of water-only fasting. However, the patients’ scores remained within the normal range, indicating that they did not exhibit symptoms of depression [59].

Interestingly, among the limited number of studies in this field, the results show discrepancies. These inconsistencies are primarily related to differences in observation duration and the fact that most studies involve very small sample sizes. Therefore, we propose that a substantial research gap exists regarding the biological effects of various self-administered fasting regimens (up to 72 h in duration) in healthy individuals, particularly those undertaken without clinical supervision.

**Table 3:** Biological effects of fasting protocols lasting 24 to 72 h in human subjects.

Fasting Time Frame	Duration	Study Design	Study Population	Positive Metabolic Outcomes	Negative Metabolic Outcomes	First Author/[Ref.]
24 h	10 weeks	Single-center, randomized controlled trial	88 females overweight females	↓ glucose, ↓ insulin, ↓ HOMA-IR, ↑ BHB	↓ insulin sensitivity, ↑ AST	Hutchison et al., 2019 [58]
24 h	Single protocol	Randomized controlled crossover trial	30 healthy cases (20 women and 10 men)	↑ GH, the observed changes in GH levels occurred independently of any associated weight loss	Not reported	Horne et al., 2025 [60]
24 h	26 weeks	Randomized controlled trial	38 adults	↓ HOMA-IR, ↓ insulin, ↓ fasting glucose, ↓ metabolic syndrome score, ↓ diastolic blood pressure, ↑ HDL-C	Not reported	Bartholomew et al., 2021 [61]
24 h	26 weeks	Randomized controlled trial	68 adults	↓ HOMA-IR, ↓ insulin, ↓ fasting glucose	↔ body weight	Horne et al., 2024 [62]
24 h	4 weeks	Randomized controlled trial	28 metabolically healthy, non-obese adults	↓ body weight, ↑ BHB, ↓ sICAM-1, ↓ systolic blood pressure, ↓ diastolic blood pressure, ↓ HR, ↓ fat: lean ratios, improved lipide profile	↓ T3	Stekovic et al., 2019 [63]
24 h	8 weeks	Randomized parallel-arm trial	8 healthy adults (4 male and 4 female)	↓ body weight, ↓ BMI, ↓ FFM	↓ SMM	Herz et al., 2024 [51]
36 h	Single protocol	Randomized crossover study	20 healthy adults (11 male and 9 female)	↑ BHB, ↓insulin, ↑ glucagon, ↓ insulin/glucagon ratio	Not reported	Deru et al., 2021 [64]
36 h	Single protocol	Controlled pilot study	20 healthy adults (10 male and 10 female)	↓ TGs, ↓glucose, ↑ circulating ketone bodies, ↑ total antioxidant capacity	Not reported	Rhodes et al., 2023 [15]
48 h	Single protocol	Randomized crossover study	11 overweight/obese women (mean age: 68.8 ± 6.4 years)	↓ body mass, ↓ BMI, ↓ FFM, ↓ glucose, ↑ ketone bodies	↑ salivary cortisol concentration, ↑ hunger, ↑ fatigue, ↑ tension, ↓ reaction time	Solianik et al., 2020 [65]
48 h	Single protocol	Paired within-subject design	16 healthy females	↓ body mass, ↓ BMI, ↔ in cortisol levels	Not reported	Mazurak et al., 2013 [66]
48 h	Single protocol	Paired within-subject design	9 healthy male participants	↓ body mass, ↓ BMI, ↓glucose, ↓ systolic blood pressure, ↓ heart rate, ↓ oxygenated hemoglobin	↑ anger, ↓ reaction time	Solianik et al., 2016 [67]
59 h	Single protocol	Controlled human study	15 young, healthy, non-obese subjects (11 men and 4 women)	↓ glucose, ↓ insulin, ↑ GH, ↑ lipolytic rate	Not reported	Goldenberg et al., 2022 [68]
60 h	Separated by a period of minimally 2 weeks.	Randomized controlled crossover study	12 healthy male participants	↓ body weight, ↓ RER, ↓ energy expenditure	Not reported	Andriessen et al., 2023 [69]
60 h	2 experimental periods	Randomized controlled crossover study	12 healthy male participants	↑ FFA, ↓ insulin, ↓ glucose, ↑ β-oxidation of FFA	↓ skeletal muscle insulin sensitivity	Hoeks et al., 2010 [70]
60 h	2 experimental periods	Randomized crossover study	10 healthy male participants	↓ leptin, ↓ chemerin, ↑ VEGF	↔ in Il-1, Il-6, TNF-α,IL-8, hs-CR, adiponectin levels	van Herpen et al., 2013 [71]
72 h	Single protocol	FAST*BRAIN study	15 healthy females	↓ body weight, ↓ glucose, ↑ ketone bodies	↑ BDI-2 score	Ding et al., 2018 [59]
72 h	Two analyses were conducted	Randomized crossover study	8 healthy males	↓ insulin, ↓ C-peptide, ↓ glucose, ↓ mTOR, ↑ FFA, ↑ glucagon, ↑ blood flow	↓ free and total T3	Vendelbo et al., 2014 [72]
72 h	Single protocol	Controlled metabolic study (lean vs. obese, pre–post design)	18 healthy men, subjects (9 normal-weight and 9 obese)	↓ insulin, ↓ C-peptide, ↓ glucose, ↑ FFA, ↑ glucagon,↔ in cortisol levels, ↓ mTOR, ↑ p62	Not reported	Bak et al., 2016 [73]

AST: aspartate transaminase; BDI-2: Beck Depression Inventory 2; BHB: beta-hydroxybutyrate; BMI: body mass index; C-peptide: connecting peptide; FFA: free fatty acids; FMM: fat-free mass; GH: growth hormone; HOMA-IR: homeostasis model assessment of insulin resistance; h: hour; HR: heart rate; HDL-C: high-density-lipoprotein cholesterol; hs-CRP: high-sensitivity C-reactive protein; IL-1: interleukin 1; IL-6: interleukin 6; IL-8: interleukin 8; mTOR: mammalian target of rapamycin; p62: adaptor protein sequestosome 1; RER: respiratory exchange ratio; sICAM-1: soluble form of intercellular adhesion molecule 1; SMM: skeletal muscle mass; TNF-α: tumor necrosis factor alpha; TGs: triglycerides; T3: triiodothyronine; VEGF: vascular endothelial growth factor; ↔: no significant change, ↓: decreased, ↑: increased.

## 3. Algorithm and Protocol for Preparing Healthy Individuals for PSTF Lasting More than 24 h and up to 72 h

**Figure 2:**
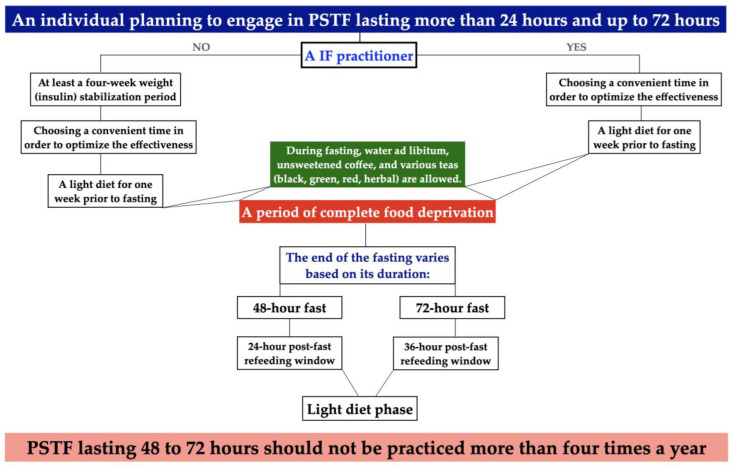
An algorithm for preparing for PSTF lasting at least 24 and up to 72 h.

We propose our own algorithm and protocol for preparing healthy individuals for PSTF lasting more than 24 h and up to 72 h (Figure 2). This protocol is based on clinical reasoning, practical experience, and established physiological principles, and is intended as a structured guide for individuals with prior IF experience as well as for less experienced volunteers. While not all components of this protocol are currently supported by direct clinical trials, the proposed approach draws upon expert consensus and selected evidence from related areas, including metabolic adaptation, hydration, the refeeding phase, and relaxation techniques aimed at mitigating potential side effects.

Volunteers who wish to undertake PSTF to achieve long-term metabolic benefits should ideally have been practicing IF for at least 6 to 12 months. During the preparation period for fasting, it is recommended to reduce the intake of simple sugars and processed foods, and to abstain from alcohol and tobacco use [74]. Such prior experience can help mitigate the risk of prematurely ending the fast or even encountering adverse effects such as headaches, disorientation, fatigue, reduced well-being, impaired concentration, and other related symptoms. However, for less experienced individuals, the preparation protocol for PSTF lasting more than 24 h and up to 72 h should begin with at least a four-week period of weight stabilization [10,14]. During this time, individuals may eat freely within their eating window (typically 8 h, following a 16 h fasting period), but during the fasting phase, only water and, occasionally, non-caloric beverages should be consumed [12]. These actions will initially help stabilize insulin secretion [10,11,37,40]. For both groups, the next step involves following a high-nutrient-density diet for one week prior to the planned fasting period that is low in carbohydrates, high in protein, and rich in green vegetables. It is also recommended to choose a time that minimizes potential distractions, such as social gatherings, work-related stress, or major life changes. This approach helps optimize the effectiveness of the fasting protocol [75]. Furthermore, during the fasting period, only specific beverages are permitted, including unlimited water, unsweetened black coffee or tea, herbal and calorie-free infusions (e.g., mint, chamomile), sugar-free electrolytes (such as sodium, magnesium, and potassium in pure form) [10,58,74,76], and certain supplements—provided they contain no calories or sugar. All other products or dietary components, including medications, may disrupt the fast.

However, a minimum water intake of 2 L per day during fasting is advised, aligning with previously published recommendations [74,77]. To support electrolyte balance, a pinch of table salt may be added to water. From a precautionary standpoint, the use of salt free of E536 (potassium ferrocyanide) and potassium iodate (KIO_3_) is advised, as these additives may exert metabolic activity, although direct evidence in the context of fasting is limited, and further studies would be needed to confirm this effect. After the fast is completed, a gradual refeeding phase—lasting at least half the duration of the fast—should follow before returning to normal dietary habits. At this stage, a high-nutrient-density diet—low in carbohydrates, high in protein, and rich in green vegetables—should be reintroduced to minimize dyspeptic symptoms, including bloating, abdominal heaviness, nausea, and vomiting [10,78].

**Figure 3:**
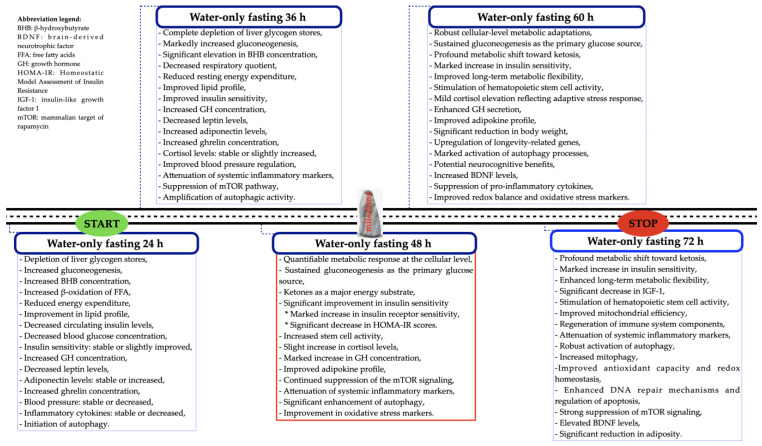
Graphical illustration of the timeframe in which measurable biological effects of PSTF occur.

Finally, based on our limited observational experience, and acknowledging the lack of robust clinical data, we suggest that PSTF lasting 48–72 h does not need to be performed more than four times a year (once per quarter) by individuals already engaged in intermittent fasting practices [14]. During fasting, individuals undertaking this challenge should anticipate certain inconveniences (intensified hunger, headaches, electrolyte imbalances, dehydration, and dizziness) [57,59], which may lead them to discontinue the fast. Therefore, before committing to a PSTF, it is important that the individual is fully aware of the real benefits of 48–72 h of PSTF (Figure 3), as well as its limitations. It is suggested to address the last issue by taking walks, and relaxation techniques like meditation and deep diaphragmatic breathing are recommended to help reduce hunger-related discomfort [79].

Despite its numerous benefits, PSTF is clearly not recommended for individuals with type 1 diabetes or eating disorders (such as bulimia or anorexia), patients receiving insulin therapy, pregnant or breastfeeding women, individuals under the age of 18 or over 75, or patients with active malignancies [74].

## 4. A Graphical Summary of the Effects of PSTF Lasting More than 24 h and up to 72 h

PSTF, defined as lasting between 24 and 72 h, is associated with numerous health benefits. These include weight loss, improvements in lipid profile, and a metabolic shift toward ketone utilization [64,65]. However, it may also induce discomfort, which often intensifies with the duration of fasting. Nevertheless, the overall benefits of PSTF appear substantial enough to outweigh its potential adverse effects. Figure 3 illustrates the temporal progression of metabolic alterations observed in individuals undergoing fasting, with detailed descriptions provided at 12 h intervals from 24 to 72 h of fasting. The diagram demonstrates that the metabolic response to fasting is clearly time-dependent. Initial alterations in the expression of proinflammatory cytokines become detectable after 24 h of fasting, with a significant reduction in their concentrations observed as the duration of fasting is extended [58,61,63]. In addition to suppressing peripheral inflammation and enhancing antioxidant defenses, fasting exerts a significant regulatory effect on autophagy through inhibition of the mTOR signaling pathway. Autophagy is typically initiated after approximately 16 h of fasting, with its activity progressively increasing as fasting duration extends, culminating in pronounced effects after 72 h [73]. Interestingly, after 48 h of fasting, significant changes become evident, including a marked increase in tissue insulin sensitivity, primarily manifested by an upregulation of insulin receptor expression. It is noteworthy that fasting improves the adipocytokine profile by increasing adiponectin secretion and reducing leptin levels. This adipocytokine pattern enhances tissue insulin sensitivity, although a significant effect becomes also apparent after 48 h of fasting [71]. Fasting lasting longer than 60 h may influence neuroplasticity through increased secretion of BDNF, a key modulator of memory, cognitive function, and mood regulation [80]. In addition to the well-documented metabolic benefits of fasting, the existing literature indicates that PSTF lasting 48–72 h also exerts positive effects on the composition and function of the gut microbiome. However, this topic falls beyond the scope of the present study.

Given the wide range of benefits associated with fasting, we recommend appropriate preparation for this intervention (Figure 2) to minimize the risk of premature termination and to maximize the health-promoting effects. However, based on Figure 3, it can be concluded that a substantial reduction in food consumption, even if periodic, markedly influences metabolic homeostasis, as well as lifespan and quality of life. The following figure summarizes key metabolic changes occurring during fasting, integrating findings from the current literature with expert interpretations of fasting physiology. Supporting references are provided here [16,54,55,56,58,60,69,71,73,77,80,81,82] for selected components such as autophagy induction, cytokine regulation, adipokine modulation, and neuroplasticity markers.

## 5. General Discussion and Future Directions

An analysis of the data presented in Table 1 and Table 2 reveals that various fasting protocols are consistently associated with a range of favorable metabolic outcomes across diverse populations. These positive effects include significant reductions in systolic and diastolic blood pressure, improved lipid profiles—particularly reductions in LDL-C, total cholesterol, and triglycerides—enhanced insulin sensitivity, and decreased markers of systemic inflammation (e.g., IL-6, TNF-α) [15,42,45,61,63]. Moreover, TRE and PSTF regimens often lead to increased levels of beneficial adipokines such as adiponectin, alongside reductions in IGF-1, a marker linked to longevity and reduced cancer risk [33,71]. A growing body of evidence suggests that IF acts as a ‘metabolic recalibration’ at the cellular and molecular level by reducing inflammation, improving hormonal balance, and increasing the efficiency of carbohydrate and lipid metabolism, ultimately helping to prevent cardiometabolic events.

The evidence strongly supports the role of TRE in improving glycemic control and mitigating risk factors associated with metabolic syndrome, particularly in overweight and obese adults [34,35]. Comparable beneficial effects on reductions in blood glucose levels and improvements in tissue insulin sensitivity have been observed with 72 h PSTF protocols [58,72,73]. Notably, several studies demonstrate that these benefits can be attained without significant losses in skeletal muscle mass or strength, a particularly critical factor in aging populations [29]. Furthermore, protocols such as 16:8 have shown high adherence rates and sustainability, highlighting their potential as long-term interventions [42].

However, it is important to acknowledge the reported adverse effects, which, although less frequently observed, are not negligible. Some studies have indicated reductions in certain hormones, such as testosterone and triiodothyronine (T3), during both IF and PSTF, which may have implications for reproductive and thyroid function in certain populations [12,32,33,50,72]. Fasting-induced reductions in testosterone levels result from complex interactions within the endocrine system, particularly involving the hypothalamic–pituitary–gonadal axis. According to Moro et al., the observed decrease in testosterone levels may be explained by leptin’s effect on the hypothalamic–pituitary–gonadal (HPG) axis [32,33]. Leptin positively regulates gonadotropin-releasing hormone (GnRH) secretion at the hypothalamic level, and its deficiency during fasting reduces luteinizing hormone (LH) and follicle-stimulating hormone (FSH) release, thereby downregulating testicular testosterone production [83]. Simultaneously, intermittent and prolonged fasting activate the hypothalamic–pituitary–adrenal axis, resulting in increased cortisol secretion. Cortisol inhibits GnRH pulsatility and directly impairs Leydig cell steroidogenesis, further exacerbating testosterone suppression [84]. This mechanism is particularly significant in prolonged fasting states, where sustained elevations in cortisol levels may compromise anabolic balance and reproductive capacity. Other hormonal factors also contribute to this suppression. Reduced insulin levels during fasting impair GnRH neuron activity, while elevated ghrelin concentrations, commonly observed during fasting, have been shown to inhibit LH secretion and testosterone biosynthesis [85]. Interestingly, Solianik et al. reported increased salivary cortisol levels during a 48 h water-only fast; however, no alterations were observed in autonomic nervous system balance, as reflected by heart rate and heart rate variability (HRV) in overweight and obese women. These findings suggest that elevated cortisol may act as a compensatory mechanism in response to prolonged hypoglycemia, reflecting broader endocrine adaptation to energy deficiency [65]. Taken together, these findings highlight the multifactorial nature of testosterone reduction in fasting states and suggest that while the metabolic benefits of fasting are notable, careful consideration should be given to its endocrine consequences, particularly in populations sensitive to androgen fluctuations.

In addition to its effects on the HPG axis, fasting also modulates other endocrine pathways, including thyroid hormone (TH) activity, which plays a pivotal role in metabolic regulation through its distinct actions on brown and white adipose tissue. In brown adipose tissue, TH stimulates thermogenesis by upregulating the expression of uncoupling proteins. In white adipose tissue, TH promotes lipolysis, increasing the release of FFAs and glycerol. While FFAs are primarily used for β-oxidation and ketone body production, glycerol can be utilized by the liver as a substrate for gluconeogenesis. Moreover, TH activates ATPases in skeletal muscle, thereby contributing to increased energy expenditure [86,87]. Stekovic et al. noted a drop in T3 levels, but no significant change in circulating thyroid-stimulating hormone and free thyroxine levels, providing evidence for preserved thyroid gland function. The authors suggested that this alteration in the thyroid axis represents a compensatory metabolic adjustment to reduced caloric intake, potentially contributing to improved health and extended lifespan [63].

Furthermore, IF induces alterations in the growth hormone (GH)/insulin-like growth factor 1 (IGF-1) axis, characterized by elevated GH levels—responsible for stimulating IGF-1 production and a concurrent significant reduction in circulating IGF-1 concentrations. Reduced IGF-1 levels have been shown to protect healthy cells, but not cancer cells, during chemotherapy, and to lower tumor incidence; they are also associated with delayed aging and decreased DNA damage [31,88]. Additionally, IF influences several key signaling pathways, including mitogen-activated protein kinases (MAPKs), Notch, and nuclear factor kappa B (NF-κB), all of which play critical roles in cellular metabolism, stress responses, and gene regulation [89]. IF can modulate the expression of components within the Notch signaling pathway in the liver, potentially conferring protection against hepatic conditions such as steatosis and fibrosis by mitigating oxidative stress and inflammation [90]. Furthermore, it helps reduce inflammatory marker levels in the hypothalamus and liver, likely through modulation of the NF-κB pathway, thereby contributing to the attenuation of systemic inflammation [91]. The fasting-induced elevation in plasma GH levels is believed to play a critical role in the regulation of whole-body substrate metabolism, primarily through the inhibition of glucose uptake and the enhancement of lipid oxidation in skeletal muscle [92,93]. Importantly, metabolic adaptations to fasting differ between lean and obese individuals, as well as between physically active and sedentary individuals, even within the same body composition category. Wijngaarden et al. [31] observed that the fasting-induced increase in plasma GH levels was significantly greater in lean individuals compared to their obese counterparts [92]. These findings may indicate impaired metabolic flexibility in individuals with obesity, potentially reflecting a diminished ability to adapt substrate utilization in response to fasting. An intriguing observation reported in the literature is that fasting may lead to a transient reduction in tissue insulin sensitivity, despite concomitant decreases in plasma insulin and glucose levels [58,70]. These fasting-related alterations in insulin sensitivity are potentially mediated by elevated plasma FFAs, occurring in the absence of hyperinsulinemia and hyperglycemia [70,71]. Moreover, fasting-induced insulin resistance does not seem to correlate with alterations in circulating proinflammatory cytokine levels. Furthermore, the authors suggested that the decline in insulin sensitivity may function as a protective mechanism, preserving glucose for the central nervous system, which relies on glucose as its primary energy source and does not depend on insulin for its uptake. Simultaneously, elevated lipid availability provides an immediately accessible energy substrate for skeletal muscle, accompanied by an increased capacity for fatty acid oxidation [71].

Fasting-induced activation of MAPK phosphatase-1 (MKP-1) plays a regulatory role in MAPK-dependent pathways involved in hepatic lipid metabolism. Nutrient deprivation stimulates hepatic MKP-1 expression, which in turn modulates the activity of specific MAPKs—such as p38 and JNK—through selective activation or downregulation across various tissues and cell types [89]. In more restrictive TRE regimens like 20/4, reports include increased sensations of hunger, elevated blood pressure, and raised LDL-C levels [46]. The LDL-C increase observed during IF may represent a physiological and temporary compensatory response to enhanced adipose tissue mobilization, and does not necessarily translate into increased cardiovascular risk, especially when accompanied by improvements in other metabolic parameters. It is well established that IF induces the exploitation of glucose and glycogen stores, leading to decreased insulin and increased glucagon levels that promote gluconeogenesis. As fasting progresses, lipolysis intensifies, releasing FFAs, which are converted via β-oxidation and used as an alternative energy source [14]. Additionally, some studies has failed to observe significant metabolic changes, indicating a possible variability in individual response or insufficient intervention duration [34,62,85].

Fasting has been shown to increase activation of the Notch signaling pathway in the hippocampus, a process associated with enhanced hippocampal neurogenesis, as indicated by elevated expression of neurotrophic factors [80]. Ketone bodies serve not only as alternative energy substrates during periods of fasting but also as potent signaling molecules that exert wide-ranging effects on cellular and organ function. They influence gene expression and modulate the activity of multiple proteins and signaling pathways involved in the regulation of longevity and age-associated physiological processes [82]. Additionally, the generation of ketone bodies, particularly BHB, during fasting provides protection against neurodegeneration and the toxin-induced nerve damage commonly observed in Parkinson’s and Alzheimer’s diseases [85]. Furthermore, BHB has been shown to inhibit the growth of certain types of cancer cells by altering their energy metabolism. BHB also supports antioxidant defenses, protects against oxidative stress, and helps prevent tissue degeneration. It regulates gene expression in part by acting as a histone deacetylase inhibitor, thereby influencing epigenetic mechanisms that control inflammation, metabolic processes, and oxidative stress [81,93].

Last but not least, suppression of the mTOR pathway is one of the key mechanisms through which intermittent and short-term fasting may exert beneficial effects. Reduced mTOR activity has been associated with diminished fat storage and adipocyte differentiation, while enhancing autophagy, cellular rejuvenation, and mitochondrial function [27,44]. These adaptations are widely regarded as beneficial for metabolic health and longevity. Specifically, fasting-induced suppression of the mTOR pathway and concomitant activation of AMP-activated protein kinase (AMPK) pathway enhances autophagic flux and may contribute to the attenuation of age-related cellular damage [94]. However, maintaining homeostasis within the mTOR signaling cascade is critical, as its chronic inhibition can lead to adverse outcomes. mTOR plays a central role in anabolic processes, including muscle protein synthesis and immune cell function; thus, prolonged suppression may contribute to sarcopenia, frailty, immunosuppression, and increased vulnerability to infections, particularly in older or chronically ill individuals [46,95,96]. Furthermore, while mTOR inhibition has therapeutic value in cancer by limiting tumor progression, it may simultaneously impair tissue regeneration in non-cancerous cells during recovery from cytotoxic therapies [97]. In metabolic terms, excessive inhibition of mTOR has been linked to glucose intolerance and insulin resistance [85,94,98], likely due to feedback dysregulation within insulin signaling cascades [98]. Bak et al. demonstrated that a 72 h fast leads to reduced mTOR phosphorylation in lean individuals, indicating a decrease in pathway activity in response to extended calorie restriction. A comparable reduction in mTOR activity was observed in obese subjects as well; however, their mTOR phosphorylation levels were consistently lower both before and after fasting compared to lean participants. These findings imply that prolonged fasting diminishes mTOR signaling in skeletal muscle regardless of body weight status, although baseline levels and the magnitude of change differ between lean and obese individuals. In obese individuals, persistently elevated nutrient availability and insulin resistance may contribute to sustained activation of the mTOR pathway, potentially impairing its responsiveness to fasting-induced metabolic adaptation [73]. However, according to Jamshed et al., an increase in mTOR gene expression was observed in the evening hours among participants following early time-restricted feeding (eTRF). The authors suggested that this upregulation may be associated with elevated insulin levels during that period, potentially indicating an adaptive physiological response to the altered feeding schedule, possibly mediated by circadian mechanisms and insulin signaling [46]. Therefore, fasting-mediated regulation of mTOR shows potential; however, it may not confer beneficial outcomes in all individuals or clinical scenarios. A balanced, context-dependent approach to mTOR regulation is essential to fully harness the therapeutic advantages of fasting without compromising physiological integrity or resilience. These findings underscore the need for personalized dietary approaches and longer-term studies, as a one-size-fits-all model may, in many cases, lead to unforeseen adverse health outcomes.

Future research should further investigate the gender-specific effects, long-term safety, and the balance between metabolic benefits and potential endocrine disruptions associated with prolonged or aggressive TRE or PSTF strategies. An important consideration is the integration of physical activity with various fasting regimens to optimize biological and cellular outcomes. The exercise does not need to be of a high intensity but should be appropriately tailored to the individual’s condition and capabilities. This strategy plays a vital role in minimizing skeletal muscle mass (SMM) loss during fasting periods. Herz et al. reported a significant reduction in SMM within the ADF group, highlighting a potential adverse effect. These results emphasize that, despite fasting’s metabolic benefits such as weight reduction, the preservation of muscle mass through a combination of adequate nutrition and individualized exercise is critical [51].

## 6. Methodology

A large-scale electronic literature search was conducted between 1 March and 31 May 2025, across major databases including PubMed (U.S. National Library of Medicine), ScienceDirect, and Google Scholar, to explore the impact of various patterns of intermittent fasting on human metabolism and associated signaling pathways. A keyword-based search strategy was employed, using the following terms: “intermittent fasting AND insulin sensitivity”, “intermittent fasting AND metabolic effect”, “intermittent fasting AND glucose metabolism”, “intermittent fasting AND lipid profile”, “intermittent fasting AND oxidative stress”, “intermittent fasting AND immune response”, “intermittent fasting AND mTOR”, “intermittent fasting AND cardiovascular risk”, “intermittent fasting AND adipokines”, “intermittent fasting AND adverse outcomes”, “time-restricted eating AND metabolic biomarkers”, “time-restricted eating AND inflammation”, “periodic short-term fasting AND metabolic health”, “periodic short-term fasting AND autophagy”, “periodic short-term fasting AND adipokines”, “periodic fasting AND insulin resistance”, “periodic short-term fasting AND human studies”, “periodic short-term fasting AND adverse outcomes”, “fasting-induced metabolic changes”, “fasting AND inflammatory markers”, “fasting protocols AND cardiometabolic risk”, “fasting duration AND metabolic adaptation”, “fasting AND endocrine regulation”, “fasting AND energy expenditure”, and “fasting AND human clinical trials”. In addition, targeted terms were used to identify literature related to specific intermittent fasting protocols, including: “OMAD AND cardiometabolic risk”, “OMAD AND appetite regulation”, “OMAD AND nutrient adequacy”, “OMAD AND adherence”, “5:2 fasting AND cardiometabolic risk”, “5:2 fasting AND appetite regulation”, “5:2 fasting AND nutrient adequacy”, “5:2 fasting AND adherence”, “ADF AND cardiometabolic risk”, “ADF AND appetite regulation”, “ADF AND nutrient adequacy”, “ADF AND adherence”. This comprehensive list of search terms was selected to ensure a broad yet focused review of the literature on various intermittent fasting regimens and their effects on metabolic health, safety, and adherence in human studies.

Inclusion Criteria

- Cross-sectional, case–control, and randomized control trials, and retrospective, prospective cohort or cohort epidemiological studies were included.
- Only studies conducted in humans were included.
- Only full-text articles in English were included.

Exclusion Criteria

- Non-human experimental models were excluded.
- Non-English papers were excluded because of the structural language barrier.
- Articles that were not peer-reviewed were excluded.
- Studies involving fasting periods for religious reasons or those exceeding 72 h were also omitted.
- Studies classified as meta-analyses or systematic reviews were excluded.

The screening of articles was conducted independently by three authors (BC, KK, PD), and all inaccuracies were identified during a final check (BC, PD). The resulting literature was then analyzed and included in our review. The papers’ assessment was based on a critical reading. Most of the included papers (85%) were published between 2015 and 2025. Notably, 63 out of 98 publications (64%), excluding 2 epidemiological sources from the WHO website (references: [5,53]), were published within the last five years. Since the review was based on previously published research, no ethical approval or patient consent was required.

## 7. Conclusions

IF and PSTF (up to 72 h) are associated with numerous beneficial health outcomes. However, the biological effects, particularly at the cellular level, are time-dependent and vary according to fasting duration. Moreover, the benefits of PSTF are significantly greater in individuals who have incorporated IF into their daily routine. Evidence also suggests that the effects of fasting are more pronounced in lean and physically active individuals due to their enhanced metabolic flexibility. The implementation of fasting protocols should be complemented by individualized physical activity interventions to mitigate the risk of muscle mass loss. Despite the strengths of various fasting protocols, there is a clear need for further studies involving larger and more diverse populations, including both healthy individuals and those with metabolic disorders, particularly in the context of self-administered PSTF (up to 72 h).

## Figures and Tables

**Table 1:** Practical summary of strengths, challenges, and protocols across different intermittent fasting regimens.

IF Regimens	Fasting/Eating Window	Recommended Daily Schedules	Intended Population	Strengths	Challenges
TRE: 16/8	16 h fasting/ 8 h feeding	10 a.m.–6 p.m. 11 a.m.–7 p.m. 12 p.m.–8 p.m.	Entry-level practitioners or Intermediate-level practitioners	Aligns with circadian rhythm; Easy to implement daily; No calorie counting required; Mitigate insulin resistance; Reduce overall adiposity; Optimize cardiometabolic parameters; Suitable for individuals across a wide age range; Supports strength training without compromising results.	This approach often involves skipping the morning meal; May not produce a clinically significant energy deficit; Can cause initial hunger or irritability during the fasting period.
TRE: 18/6	18 h fasting/ 6 h feeding	12 p.m.–6 p.m. 1 p.m.–7 p.m. 2 p.m.–8 p.m.	Intermediate-level practitioners	Greater metabolic effect; No calorie counting required; Promotes weight loss; Allows for two balanced meals, making nutrient intake more manageable.	Higher level of restrictions; May not provide as profound effects on autophagy as longer fasts; Possible hunger, irritability, or concentration issues during fasting, particularly in beginners.
TRE: 20/4	20 h fasting/ 4 h feeding	2 p.m.–6 p.m. 3 p.m.–7 p.m. 4 p.m.–8 p.m.	Advanced practitioners	Large calorie reduction; No calorie counting required; Supports healthy weight management; Suitable for individuals with prior experience in intermittent fasting.	High level of restrictions; Difficult to maintain long-term; Risk of nutrient deficiencies if meals are not sufficiently nutrient-dense; Not suitable for pregnant or breastfeeding women and eating disorders individuals, and T1DM; Emotional variability.
One meal a day (OMAD)	23 h fasting/1 h feeding	One meal selected, e.g., dinner	Advanced practitioners	Large calorie reduction; No calorie counting required; Stronger metabolic effects; Potential to support autophagy and mitochondrial function; Simplifies meal planning and reduces time spent eating and preparing meals.	Difficult to maintain long-term; Risk of excessive hunger or appetite following the fasting period; Risk of nutrient deficiencies if meals are not sufficiently nutrient-dense; Not suitable for pregnant or breastfeeding women and eating disorders individuals, and T1DM; Emotional variability.
5:2	5 days normal feeding/ 2 days 500–600 kcal	Freely	Intermediate-level practitioners	High flexibility; No calorie counting required; Suitable for individuals with T2DM; May improve cardiovascular hemodynamics; Optimizes cardiometabolic parameters; Appropriate for a wide range of individuals, including those who prefer less restrictive fasting protocols.	Slower onset of effects; Risk of overeating on non-fasting days, potentially offsetting benefits; Not suitable for pregnant or breastfeeding women, individuals with eating disorders, or those with T1DM.
ADF	Every other day fasting 0–500 kcal	Day 1: Normal Day 2: Fasting	Advanced practitioners	Strong metabolic effects (e.g., promotes autophagy and cellular repair); Optimizes cardiometabolic parameters; Clearly structured regimen, which may support adherence; Does not require daily calorie restriction, allowing flexibility on feeding days.	Difficult to adhere to long-term; Inconsistent fasting stimulus may limit metabolic adaptation; Risk of overeating on non-fasting days, potentially offsetting benefits; Emotional variability.

**Table 2:** Biological effects of various TRE protocols in human subjects.

Fasting/Eating Time	Duration	Study Design	Study Population	Positive Metabolic Outcomes	Negative Metabolic Outcomes	First Author, Year [Ref.]
14/10	12 weeks	Single-arm, paired-sample trial	19 participants with metabolic syndrome	↓ systolic and diastolic blood pressure, ↓ TC, ↓ LDL-C, ↓ non-HDL-C, ↓ HbA1c	Not reported	Wilkinson et al., 2020 [35]
14/10	12 weeks	Randomized controlled trial	120 overweight adults with type 2 diabetes	↓ HbA1c, ↓ glucose, ↑ insulin sensitivity, ↓ TGs, ↓ TC, ↓ LDL-C	Not reported	Che et al., 2021 [36]
15/9	1 week	Randomized crossover trial	15 adults with prediabetes	↓ TGs, ↑ glucose responses	Not reported	Hutchison et al., 2019 [37]
16/8	8 weeks	Single-blind randomized study	34 resistance-trained males	↑ adiponectin, ↓ total leptin, ↓ IGF-1	↓ testosterone, ↓ T3	Moro et al., 2016 [33]
16/8	12 months	Single-blind randomized study	20 healthy resistance-trained subjects	↓ IL-6, ↓ IL-1β, ↑ HDL-C, ↓ LDL-C, ↓ TNF-α, ↓ IGF-1	↓ testosterone	Moro et al., 2021 [32]
16/8	4 weeks	Single-blind randomized study	16 elite under-23 cyclists	↑ adiponectin, ↓ IGF-1	↓ free testosterone	Moro et al., 2020 [25]
16/8	4 weeks	Pilot, single-arm interventional study	10 overweight older adults	↓ IL-1β, ↓ TNF-α	Not reported	Ezzati et al., 2025 [38]
16/8	4 weeks	Randomized crossover trial	17 adults with obesity	Not reported	Not reported	Mena-Hernández et al., 2024 [39]
16/8	12 weeks	Quasi-experimental trial	62 menopausal women	↓ glucose, ↓ insulin, ↓ HOMA-IR	Not reported	Jóźwiak et al., 2024 [40]
16/8	4 weeks	Randomized pre-post pilot study	22 physically active men	↑ adiponectin, ↑ HDL-C	Not reported	McAllister et al., 2020 [41]
16/8	12 weeks	Randomized controlled trial	46 adults with obesity	↓ systolic blood pressure	Not reported	Gabel et al., 2018 [42]
16/8	12 months	Randomized controlled trial	90 adults with obesity	↔ in total testosterone, DHEA, SHBG, estradiol, and progesterone	Not reported	Lin et al., 2023 [43]
16/8	2 weeks	Randomized controlled trial	10 obese females	↓ mTOR, ↓ fat mass, ↓ body weight	Not reported	Rejeki et al., 2024 [44]
18/6	12 weeks	Randomized clinical trial	116 adults with BMI from 27 to 43 kg/m^2^	Not reported	↔ in fasting glucose, fasting insulin, HOMA-IR, HbA_1C_, TGs, TC, LDL-C or HDL-C	Lowe et al., 2020 [28]
18/6	5 weeks	Supervised controlled feeding trial	8 men with prediabetes	↑ insulin sensitivity, ↑ β cell responsiveness, ↓ blood pressure, ↓ oxidative stress levels	Not reported	Sutton et al., 2018 [45]
18/6	12 weeks	Randomized crossover trial	11 overweight adults	↑ glycemic control, ↓ glycemic excursions, ↑ ketone levels, ↓ cortisol levels, ↑ BDNF, ↑ sirtuine	↑ mTOR	Jamshed et al., 2019 [46]
18/6	4 days	Randomized crossover trial	11 overweight adults	↓ levels of active ghrelin, ↓ leptin, ↓ GLP-1	Not reported	Ravussin et al., 2019 [29]
18/6	4 weeks	Randomized controlled trial	25 healthy adults	Not reported	↔ in systolic blood pressure, fasting plasma glucose and LDL-C	Mayra et al., 2022 [30]
20/4 18/6	8 weeks	Randomized controlled trial	23 premenopausal and postmenopausal women	↓ DHEA	↔ in testosterone, androstenedione, SHBG, estradiol, estrone, and progesterone	Kalam et al., 2023 [47]
20/4	8 weeks	Randomized controlled trial	15 normal-weight adults	↑ HDL-C, ↓ cortisol	↑ hunger, ↑ blood pressure, ↑ LDL-C	Stote et al., 2007 [48]
20/4 18/6	8 weeks	Randomized controlled trial	49 obese adults	↓ fasting glucose, ↓ insulin, ↓ oxidative stress levels	↔ in TNF-α, IL-6, TGs, TC, LDL-C or HDL-C	Cienfuegos et al., 2020 [34]
20/4	16 weeks	Randomized crossover trial	15 normal-weight adults	Not reported	↑ glucose, delayed insulin response, ↑ ghrelin, ↔ differences in levels of insulin, leptin, adiponectin, resistin, and BDNF	Carlson et al., 2007 [49]
20/4	2 weeks	Randomized crossover trial	8 normal-weight, healthy adults	↑ GSK, ↓ mTOR	↔ in insulin-mediated peripheral glucose uptake, hepatic insulin sensitivity, insulin sensitivity of adipose tissue, or proteolysis	Soeters et al., 2009 [50]
20/4 18/6	8 weeks	Randomized parallel-arm trial	35 adults with obesity	↓ fat mas, ↓ lean mass, ↓ visceral fat mass, ↓ waist circumference, ↓ insulin, ↓ HOMA-IR, ↓ HbA1c,↓ 8-isoprostane	↔ in circulating levels of IGF-1, IGFBP1, and IGFBP3	Akasheh et al., 2024 [12]
20/4 18/6	8 weeks	Randomized parallel-arm trial	17 healthy adults	↓ WHR	↑ LDL-C, ↔ in body weight or BMI	Herz et al., 2024 [51]
^	10 weeks	Pilot feasibility study	13 adults with obesity	↓ fat mass, ↓ fasting glucose	Not reported	Antoni et al., 2018 [52]

BNDF: brain-derived neurotrophic factor; DHEA: dehydroepiandrosterone; GLP-1: glucagon-like peptide-1; GSK: glycogen synthase kinase; HbA1c: hemoglobine A1c; HDL-C: high-density-lipoprotein cholesterol; HOMA-IR: homeostasis model assessment of insulin resistance; IL-1: Interleukin 1; IL-6: Interleukin 6; IGF-1: insulin-like growth factor 1; IGFBP1: insulin-like growth factor binding proteins 1; IGFBP3: insulin-like growth factor binding proteins 3; LDL-C: low-density-lipoprotein cholesterol; mTOR: mammalian target of rapamycin; SHBG: sex hormone-binding globulin; TC: total cholesterol; TNF-α: tumor necrosis factor alpha; TGs: triglycerides; T3: triiodothyronine; WHR: waist-to-hip-ratio, ^: TRF group’s delayed first energy intake of the day and early last energy intake of the day, delayed and early by 1·5 h, compared with their dietary patterns calculated from the baseline diaries; ↔: no significant change, ↓: decreased, ↑: increased.

## Abbreviations

The following abbreviations are used in this manuscript:

ADF	Alternate-day fasting
AKT	Protein kinase B
AST	Aspartate transaminase
BDI-2	Beck Depression Inventory-2
BDN	Brain-derived neurotrophic factor
BHB	Beta-hydroxybutyrate
DHEA	Dehydroepiandrosterone
FFA	Free fatty acids
FSH	Follicle-stimulating hormone
GH	Growth hormone
GnRH	Gonadotropin-releasing hormone
GSK	Glycogen synthase kinase
HbA1c	Hemoglobine A1c
HDL-C	High-density-lipoprotein cholesterol
HLD	Hyperlipidemia
HOMA-IR	Homeostasis Model Assessment of Insulin Resistance
HTN	Hypertension
IF	Intermittent fasting
IGF-1	Insulin-like growth factor type 1
IGFBP-1	Insulin-like growth factor binding proteins 1
IGFBP-2	Insulin-like growth factor binding proteins 2
IL-1	Interleukin 1
IL-6	Interleukin 6
IL-8	Interleukin 8
LDL-C	Low-density-lipoprotein cholesterol
LH	Luteinizing hormone
MAPKs	Mitogen-activated protein kinases
MASLD	Metabolic dysfunction-associated steatotic liver disease
MKP-1	Mitogen-activated protein kinase phosphatase 1
mTOR	Mammalian target of rapamycin
NF-κB	Nuclear factor kappa B
PTSF	Periodic short-term fasting
RER	Respiratory exchange ratio
ROS	Reactive oxygen species
SHBG	Sex hormone-binding globulin
SOD	Superoxide dismutase
T1DM	Type 1 diabetes mellitus
T2DM	Type 2 diabetes mellitus
T3	Triiodothyronine
TC	Total cholesterol
TGs	Triglycerides
TNF-α	Tumor necrosis factor alpha
TRE	Time-restricted eating
WHO	World Health Organization

## References

[1] Thompson S., Madsen L.T., Bazzell A. (2023). Impact of Fasting on Patients with Cancer: An Integrative Review. J. Adv. Pract. Oncol..

[2] Zhang H., Zhou X.D., Shapiro M.D., Lip G.Y.H., Tilg H., Valenti L., Somers V.K., Byrne C.D., Targher G., Yang W. (2024). Global burden of metabolic diseases, 1990–2021. Metabolism.

[3] Chew N.W.S., Ng C.H., Tan D.J.H., Kong G., Lin C., Chin Y.H., Lim W.H., Huang D.Q., Quek J., Fu C.E. (2023). The global burden of metabolic disease: Data from 2000 to 2019. Cell Metab..

[4] Hafizi N.A., Azhari N.S., Ali A., Zakaria N.S., Yusof H.M. (2023). Outcomes of different types of intermittent fasting for practitioners in terms of nutritional status and quality of life: A systematic review. J. Appl. Pharm. Sci..

[5] https://www.who.int/news-room/fact-sheets/detail/obesity-and-overweight.

[6] López-Tenorio I.I., Aguilar-Villegas Ó.R., Espinoza-Palacios Y., Segura-Real L., Peña-Aparicio B., Amedei A., Aguirre-García M.M. (2024). Primary Prevention Strategy for Non-Communicable Diseases (NCDs) and Their Risk Factors: The Role of Intestinal Microbiota. Biomedicines.

[7] Gassner L., Zechmeister-Koss I., Reinsperger I. (2022). National Strategies for Preventing and Managing Non-communicable Diseases in Selected Countries. Front. Public Health.

[8] Xu X., Wang L., Zhang K., Zhang Y., Fan G. (2023). Managing metabolic diseases: The roles and therapeutic prospects of herb-derived polysaccharides. Biomed. Pharmacother..

[9] Folin O., Denis W. (1915). On starvation and obesity with special regerence to acidosis. J. Biol. Chem..

[10] Visioli F., Mucignat-Caretta C., Anile F., Panaite S.A. (2022). Traditional and Medical Applications of Fasting. Nutrients.

[11] Nye K., Cherrin C., Meires J. (2024). Intermittent Fasting: Exploring Approaches, Benefits, and Implications for Health and Weight Management. J. Nurse Pract..

[12] Akasheh R.T., Ankireddy A., Gabel K., Ezpeleta M., Lin S., Tamatam C.M., Reddy S.P., Spring B., Cheng T.-Y.D., Fontana L. (2024). Effect of Time-Restricted Eating on Circulating Levels of IGF1 and Its Binding Proteins in Obesity: An Exploratory Analysis of a Randomized Controlled Trial. Nutrients.

[13] Hong B.V., Rhodes C.H., Agus J.K., Tang X., Zhu C., Zheng J.J., Zivkovic A.M. (2023). A single 36-h water-only fast vastly remodels the plasma lipidome. Front. Cardiovasc. Med..

[14] Longo V.D., Di Tano M., Mattson M.P., Guidi N. (2021). Intermittent and periodic fasting, longevity and disease. Nat. Aging.

[15] Rhodes C.H., Zhu C., Agus J., Tang X., Li Q., Engebrecht J., Zivkovic A.M. (2023). Human fasting modulates macrophage function and upregulates multiple bioactive metabolites that extend lifespan in Caenorhabditis elegans: A pilot clinical study. Am. J. Clin. Nutr..

[16] Koppold D.A., Breinlinger C., Hanslian E., Kessler C., Cramer H., Khokhar A.R., Peterson C.M., Tinsley G., Vernieri C., Bloomer R.J. (2024). International consensus on fasting terminology. Cell Metab..

[17] Antoni R., Johnston K.L., Collins A.L., Robertson M.D. (2018). Intermittent v. continuous energy restriction: Differential effects on postprandial glucose and lipid metabolism following matched weight loss in overweight/obese participants. Br. J. Nutr..

[18] Cruces-Sande M., Arcones A.C., Vila-Bedmar R., Val-Blasco A., Sharabi K., Díaz-Rodríguez D., Puigserver P., Mayor F., Murga C. (2020). Autophagy mediates hepatic GRK2 degradation to facilitate glucagon-induced metabolic adaptation to fasting. FASEB J..

[19] Sui X., Wang H., Wu F., Yang C., Zhang H., Xu Z., Guo Y., Guo Z., Xin B., Ma T. (2022). Hepatic metabolite responses to 4-day complete fasting and subsequent refeeding in rats. PeerJ.

[20] Domaszewski P., Konieczny M., Pakosz P., Łukaniszyn-Domaszewska K., Mikuláková W., Sadowska-Krępa E., Anton S. (2022). Effect of a six-week times restricted eating intervention on the body composition in early elderly men with overweight. Sci. Rep..

[21] Katsarou A.L., Katsilambros N.L., Koliaki C.C. (2021). Intermittent Energy Restriction, Weight Loss and Cardiometabolic Risk: A Critical Appraisal of Evidence in Humans. Healthcare.

[22] Domaszewski P., Konieczny M., Pakosz P., Baczkowicz D., Sadowska-Krępa E. (2020). Effect of a six-week intermittent fasting intervention program on the composition of the human body in women over 60 years of age. Int. J. Environ. Res. Public Health.

[23] Domaszewski P., Rogowska A.M. (2024). Examining Associations Between Fasting Behavior, Orthorexia Nervosa, and Eating Disorders. Nutrients.

[24] Domaszewski P., Konieczny M., Dybek T., Łukaniszyn-Domaszewska K., Anton S., Sadowska-Krępa E., Skorupska E. (2023). Comparison of the effects of six-week time-restricted eating on weight loss, body composition, and visceral fat in overweight older men and women. Exp. Gerontol..

[25] Moro T., Tinsley G., Longo G., Grigoletto D., Bianco A., Ferraris C., Guglielmetti M., Veneto A., Tagliabue A., Marcolin G. (2020). Time-restricted eating effects on performance, immune function, and body composition in elite cyclists: A randomized controlled trial. J. Int. Soc. Sports Nutr..

[26] Drummond M.D.M., Soares P.S.G., Savoi L.A., Silva R.A.D. (2024). Fasting reduces satiety and increases hunger but does not affect the performance in resistance training. Biol. Sport.

[27] Shabkhizan R., Haiaty S., Moslehian M.S., Bazmani A., Sadeghsoltani F., Saghaei Bagheri H., Rahbarghazi R., Sakhinia E. (2023). The Beneficial and Adverse Effects of Autophagic Response to Caloric Restriction and Fasting. Adv. Nutr..

[28] Lowe D.A., Wu N., Rohdin-Bibby L., Moore A.H., Kelly N., Liu Y.E., Philip E., Vittinghoff E., Heymsfield S.B., Olgin J.E. (2020). Effects of Time-Restricted Eating on Weight Loss and Other Metabolic Parameters in Women and Men with Overweight and Obesity: The TREAT Randomized Clinical Trial. JAMA Intern. Med..

[29] Ravussin E., Beyl R.A., Poggiogalle E., Hsia D.S., Peterson C.M. (2019). Early Time-Restricted Feeding Reduces Appetite and Increases Fat Oxidation But Does Not Affect Energy Expenditure in Humans. Obesity.

[30] Mayra S.T., Chondropoulos K., De Leon A., Kravat N., Johnston C.S. (2022). The feasibility and preliminary efficacy of early time-restricted eating on diet quality in college students: A randomized study. Obes. Res. Clin. Pract..

[31] Salvadori G., Mirisola M.G., Longo V.D. (2021). Intermittent and Periodic Fasting, Hormones, and Cancer Prevention. Cancers.

[32] Moro T., Tinsley G., Pacelli F.Q., Marcolin G., Bianco A., Paoli A. (2021). Twelve Months of Time-restricted Eating and Resistance Training Improves Inflammatory Markers and Cardiometabolic Risk Factors. Med. Sci. Sports Exerc..

[33] Moro T., Tinsley G., Bianco A., Marcolin G., Pacelli Q.F., Battaglia G., Palma A., Gentil P., Neri M., Paoli A. (2016). Effects of eight weeks of time-restricted feeding (16/8) on basal metabolism, maximal strength, body composition, inflammation, and cardiovascular risk factors in resistance-trained males. J. Transl. Med..

[34] Cienfuegos S., Gabel K., Kalam F., Ezpeleta M., Wiseman E., Pavlou V., Lin S., Oliveira M.L., Varady K.A. (2020). Effects of 4- and 6-h Time-Restricted Feeding on Weight and Cardiometabolic Health: A Randomized Controlled Trial in Adults with Obesity. Cell Metab..

[35] Wilkinson M.J., Manoogian E.N.C., Zadourian A., Lo H., Fakhouri S., Shoghi A., Wang X., Fleischer J.G., Navlakha S., Panda S. (2020). Ten-Hour Time-Restricted Eating Reduces Weight, Blood Pressure, and Atherogenic Lipids in Patients with Metabolic Syndrome. Cell Metab..

[36] Che T., Yan C., Tian D., Zhang X., Liu X., Wu Z. (2021). Time-restricted feeding improves blood glucose and insulin sensitivity in overweight patients with type 2 diabetes: A randomised controlled trial. Nutr. Metab..

[37] Hutchison A.T., Regmi P., Manoogian E.N.C., Fleischer J.G., Wittert G.A., Panda S., Heilbronn L.K. (2019). Time-Restricted Feeding Improves Glucose Tolerance in Men at Risk for Type 2 Diabetes: A Randomized Crossover Trial. Obesity.

[38] Ezzati A., Tamargo J.A., Golberg L., Haub M.D., Anton S.D. (2025). The Effects of Time-Restricted Eating on Inflammation and Oxidative Stress in Overweight Older Adults: A Pilot Study. Nutrients.

[39] Mena-Hernández D.R., Jiménez-Domínguez G., Méndez J.D., Olvera-Hernández V., Martínez-López M.C., Guzmán-Priego C.G., Reyes-López Z., Ramos-García M., Juárez-Rojop I.E., Zavaleta-Toledo S.S. (2024). Effect of Early Time-Restricted Eating on Metabolic Markers and Body Composition in Individuals with Overweight or Obesity. Nutrients.

[40] Jóźwiak B., Domin R., Krzywicka M., Laudańska-Krzemińska I. (2024). Effect of exercise alone and in combination with time-restricted eating on cardiometabolic health in menopausal women. J. Transl. Med..

[41] McAllister M.J., Pigg B.L., Renteria L.I., Waldman H.S. (2020). Time-restricted feeding improves markers of cardiometabolic health in physically active college-age men: A 4-week randomized pre-post pilot study. Nutr. Res..

[42] Gabel K., Hoddy K.K., Haggerty N., Song J., Kroeger C.M., Trepanowski J.F., Panda S., Varady K.A. (2018). Effects of 8-h time restricted feeding on body weight and metabolic disease risk factors in obese adults: A pilot study. Nutr. Health Aging.

[43] Lin S., Cienfuegos S., Ezpeleta M., Pavlou V., Chakos K., McStay M., Runchey M.C., Alexandria S.J., Varady K.A. (2023). Effect of Time-Restricted Eating versus Daily Calorie Restriction on Mood and Quality of Life in Adults with Obesity. Nutrients.

[44] Rejeki P.S., Pranoto A., Widiatmaja D.M., Utami D.M., Izzatunnisa N., Sugiharto, Lesmana R., Halim S. (2024). Combined Aerobic Exercise with Intermittent Fasting Is Effective for Reducing mTOR and Bcl-2 Levels in Obese Females. Sports.

[45] Sutton E.F., Beyl R., Early K.S., Cefalu W.T., Ravussin E., Peterson C.M. (2018). Early Time-Restricted Feeding Improves Insulin Sensitivity, Blood Pressure, and Oxidative Stress Even without Weight Loss in Men with Prediabetes. Cell Metab..

[46] Jamshed H., Beyl R.A., Manna D.L.D., Yang E.S., Ravussin E., Peterson C.M. (2019). Early Time-Restricted Feeding Improves 24-Hour. Nutrients.

[47] Kalam F., Akasheh R.T., Cienfuegos S., Ankireddy A., Gabel K., Ezpeleta M., Lin S., Tamatam C.M., Reddy S.P., Spring B. (2023). Effect of time-restricted eating on sex hormone levels in premenopausal and postmenopausal females. Obesity.

[48] Stote K.S., Baer D.J., Spears K., Paul D.R., Harris G.K., Rumpler W.V., Strycula P., Najjar S.S., Ferrucci L., Ingram D.K. (2007). A controlled trial of reduced meal frequency without caloric restriction in healthy, normal-weight, middle-aged adults. Am. J. Clin. Nutr..

[49] Carlson O., Martin B., Stote K.S., Golden E., Maudsley S., Najjar S.S., Ferrucci L., Ingram D.K., Longo D.L., Rumpler W.V. (2007). Impact of reduced meal frequency without caloric restriction on glucose regulation in healthy, normal-weight middle-aged men and women. Metabolism.

[50] Soeters M.R., Lammers N.M., Dubbelhuis P.F., Ackermans M.T., Jonkers-Schuitema C.F., Fliers E., Sauerwein H.P., Aerts J.M., Serlie M.J. (2009). Intermittent fasting does not affect whole-body glucose, lipid, or protein metabolism. Am. J. Clin. Nutr..

[51] Herz D., Karl S., Weiß J., Zimmermann P., Haupt S., Zimmer R.T., Schierbauer J., Wachsmuth N.B., Erlmann M.P., Niedrist T. (2024). Effects of Different Types of Intermittent Fasting Interventions on Metabolic Health in Healthy Individuals (EDIF): A Randomised Trial with a Controlled-Run in Phase. Nutrients.

[52] Antoni R., Robertson T.M., Robertson M.D., Johnston J.D. (2018). A pilot feasibility study exploring the effects of a moderate time-restricted feeding intervention on energy intake, adiposity and metabolic physiology in free-living human subjects. J. Nutr. Sci..

[53] https://www.who.int/news/item/24-07-2024-hunger-numbers-stubbornly-high-for-three-consecutive-years-as-global-crises-deepen--un-report.

[54] Parcha V., Heindl B., Kalra R., Li P., Gower B., Arora G., Arora P. (2022). Insulin Resistance and Cardiometabolic Risk Profile Among Nondiabetic American Young Adults: Insights From NHANES. J. Clin. Endocrinol. Metab..

[55] Fahed M., Abou Jaoudeh M.G., Merhi S., Mosleh J.M.B., Ghadieh R., Al Hayek S., El Hayek Fares J.E. (2020). Evaluation of risk factors for insulin resistance: A cross sectional study among employees at a private university in Lebanon. BMC Endocr. Disord..

[56] Ogłodek E., Pilis W. (2021). Is Water-Only Fasting Safe?. Glob. Adv. Health Med..

[57] Wilhelmi de Toledo F., Grundler F., Bergouignan A., Drinda S., Michalsen A. (2019). Safety, health improvement and well-being during a 4 to 21-day fasting period in an observational study including 1422 subjects. PLoS ONE.

[58] Hutchison A.T., Liu B., Wood R.E., Vincent A.D., Thompson C.H., O’Callaghan N.J., Wittert G.A., Heilbronn L.K. (2019). Effects of Intermittent Versus Continuous Energy Intakes on Insulin Sensitivity and Metabolic Risk in Women with Overweight. Obesity.

[59] Ding X.Q., Maudsley A.A., Schweiger U., Schmitz B., Lichtinghagen R., Bleich S., Lanfermann H., Kahl K.G. (2018). Effects of a 72 h fasting on brain metabolism in healthy women studied in vivo with magnetic resonance spectroscopic imaging. J. Cereb. Blood Flow. Metab..

[60] Horne B.D., Anderson J.L., May H.T., Bair T.L., Le V.T., Iverson L., Knowlton K.U., Muhlestein J.B. (2025). Weight loss-independent changes in human growth hormone during water-only fasting: A secondary evaluation of a randomized controlled trial. Front. Endocrinol..

[61] Bartholomew C., Muhlestein J.B., May H.T., Le V.T., Galenko O., Garrett K.D., Brunker C., Hopkins R.O., Carlquist J.F., Knowlton K.U. (2021). Randomized controlled trial of once-per-week intermittent fasting for health improvement: The WONDERFUL trial. Eur. Heart J. Open.

[62] Horne B.D., Anderson J.L., May H.T., Bair T.L., Le V.T., Iverson L., Knowlton K.U., Muhlestein J.B. (2024). Insulin resistance reduction, intermittent fasting, and human growth hormone: Secondary analysis of a randomized trial. NPJ Metab. Health Dis..

[63] Stekovic S., Hofer S.J., Tripolt N., Aon M.A., Royer P., Pein L., Stadler J.T., Pendl T., Prietl B., Url J. (2019). Alternate Day Fasting Improves Physiological and Molecular Markers of Aging in Healthy, Non-obese Humans. Cell Metab..

[64] Deru L.S., Bikman B.T., Davidson L.E., Tucker L.A., Fellingham G., Bartholomew C.L., Yuan H.L., Bailey B.W. (2021). The Effects of Exercise on β-Hydroxybutyrate Concentrations over a 36-h Fast: A Randomized Crossover Study. Med. Sci. Sports Exerc..

[65] Solianik R., Žlibinaitė L., Drozdova-Statkevičienė M., Sujeta A. (2020). Forty-eight-hour fasting declines mental flexibility but improves balance in overweight and obese older women. Physiol. Behav..

[66] Mazurak N., Günther A., Grau F.S., Muth E.R., Pustovoyt M., Bischoff S.C., Zipfel S., Enck P. (2013). Effects of a 48-h fast on heart rate variability and cortisol levels in healthy female subjects. Eur. J. Clin. Nutr..

[67] Solianik R., Sujeta A., Terentjevienė A., Skurvydas A. (2016). Effect of 48 h Fasting on Autonomic Function, Brain Activity, Cognition, and Mood in Amateur Weight Lifters. Biomed. Res. Int..

[68] Goldenberg N., Horowitz J.F., Gorgey A., Sakharova A., Barkan A.L. (2022). Role of pulsatile growth hormone (GH) secretion in the regulation of lipolysis in fasting humans. Clin. Diabetes Endocrinol..

[69] Andriessen C., Doligkeit D., Moonen-Kornips E., Mensink M., Hesselink M.K.C., Hoeks J., Schrauwen P. (2023). The impact of prolonged fasting on 24h energy metabolism and its 24h rhythmicity in healthy, lean males: A randomized cross-over trial. Clin. Nutr..

[70] Hoeks J., van Herpen N.A., Mensink M., Moonen-Kornips E., van Beurden D., Hesselink M.K., Schrauwen P. (2010). Prolonged fasting identifi es skeletal muscle mitochondrial dysfunction as consequence rather than cause of human insulin resistance. Diabetes.

[71] van Herpen N.A., Sell H., Eckel J., Schrauwen P., Mensink R.P. (2013). Prolonged fasting and the effects on biomarkers of inflammation and on adipokines in healthy lean men. Horm. Metab. Res..

[72] Vendelbo M.H., Møller A.B., Christensen B., Nellemann B., Clasen B.F., Nair K.S., Jørgensen J.O., Jessen N., Møller N. (2014). Fasting increases human skeletal muscle net phenylalanine release and this is associated with decreased mTOR signaling. PLoS ONE.

[73] Bak A.M., Møller A.B., Vendelbo M.H., Nielsen T.S., Viggers R., Rungby J., Pedersen S.B., Jørgensen J.O., Jessen N., Møller N. (2016). Differential regulation of lipid and protein metabolism in obese vs. lean subjects before and after a 72-h fast. Am. J. Physiol. Endocrinol. Metab..

[74] Attinà A., Leggeri C., Paroni R., Pivari F., Dei Cas M., Mingione A., Dri M., Marchetti M., Di Renzo L. (2021). Fasting: How to Guide. Nutrients.

[75] Zeiler E., Gabriel S., Ncube M., Thompson N., Newmire D., Scharf E.L., Goldhamer A.C., Myers T.R. (2024). Prolonged Water-Only Fasting Followed by a Whole-Plant-Food Diet Is a Potential Long-Term Management Strategy for Hypertension and Obesity. Nutrients.

[76] Sciarrillom C.M., Keirns B.H., Elliott D.C., Emerson S.R. (2021). The effect of black coffee on fasting metabolic markers and an abbreviated fat tolerance test. Clin. Nutr. ESPEN.

[77] Armstrong L.E., Johnson E.C. (2018). Water Intake, Water Balance, and the Elusive DailyWater Requirement. Nutrients.

[78] Longo V.D., Mattson M.P. (2014). Fasting: Molecular mechanisms and clinical applications. Cell Metab..

[79] Torske A., Bremer B., Hölzel B.K., Maczka A., Koch K. (2024). Mindfulness meditation modulates stress-eating and its neural correlates. Sci. Rep..

[80] Baik S.H., Rajeev V., Fann D.Y., Jo D.G., Arumugam T.V. (2020). Intermittent fasting increases adult hippocampal neurogenesis. Brain Behav..

[81] Nencioni A., Caffa I., Cortellino S., Longo V.D. (2018). Fasting and cancer: Molecular mechanisms and clinical application. Nat. Rev. Cancer.

[82] de Cabo R., Mattson M.P. (2019). Effects of Intermittent Fasting on Health, Aging, and Disease. N. Engl. J. Med..

[83] Ahlma R.S., Prabakaran D., Mantzoros C., Qu D., Lowell B., Maratos-Flier E., Flier J.S. (1996). Role of leptin in the neuroendocrine response to fasting. Nature.

[84] Fernández-Fernández R., Tena-Sempere M., Navarro V.M., Barreiro M.L., Castellano J.M., Aguilar E., Pinilla L. (2006). Effects of ghrelin upon gonadotropin-releasing hormone and gonadotropin secretion in adult female rats: In vivo and in vitro studies. Neuroendocrinology.

[85] Panwar V., Singh A., Bhatt M., Tonk R.K., Azizov S., Raza A.S., Sengupta S., Kumar D., Garg M. (2023). Multifaceted role of mTOR (mammalian target of rapamycin) signaling pathway in human health and disease. Signal Transduct. Target. Ther..

[86] Mullur R., Liu Y.Y., Brent G.A. (2014). Thyroid hormone regulation of metabolism. Physiol. Rev..

[87] Kim B.H., Joo Y., Kim M.S., Choe H.K., Tong Q., Kwon O. (2021). Effects of Intermittent Fasting on the Circulating Levels and Circadian Rhythms of Hormones. Endocrinol. Metab..

[88] Riedinger C.J., Kimball K.J., Kilgore L.C., Bell C.W., Heidel R.E., Boone J.D. (2020). Water only fasting and its effect on chemotherapy administration in gynecologic malignancies. Gynecol. Oncol..

[89] Sellers J., Brooks A., Fernando S., Westenberger G., Junkins S., Smith S., Min K., Lawan A. (2021). Fasting-Induced Upregulation of MKP-1 Modulates the Hepatic Response to Feeding. Nutrients.

[90] Allahverdi H. (2024). Exploring the therapeutic potential of plasma from intermittent fasting and untreated rats on aging-induced liver damage. J. Cell Mol. Med..

[91] Yang W., Cao M., Mao X., Wei X., Li X., Chen G., Zhang J., Wang Z., Shi J., Huang H. (2016). Alternate-day fasting protects the livers of mice against high-fat diet-induced inflammation associated with the suppression of Toll-like receptor 4/nuclear factor κB signaling. Nutr. Res..

[92] Wijngaarden M.A., van der Zon G.C., van Dijk K.W., Pijl H., Guigas B. (2013). Effects of prolonged fasting on AMPK signaling, gene expression, and mitochondrial respiratory chain content in skeletal muscle from lean and obese individuals. Am. J. Physiol. Endocrinol. Metab..

[93] Newman J.C., Verdin E. (2014). β-hydroxybutyrate: Much more than a metabolite. Diabetes Res. Clin. Pract..

[94] Kalam F., James D.L., Li Y.R., Coleman M.F., Kiesel V.A., Cespedes Feliciano E.M., Hursting S.D., Sears D.D., Kleckner A.S. (2023). Intermittent fasting interventions to leverage metabolic and circadian mechanisms for cancer treatment and supportive care outcomes. J. Natl. Cancer Inst. Monogr..

[95] Saxton R.A., Sabatini D.M. (2017). mTOR Signaling in Growth, Metabolism, and Disease. Cell.

[96] Yecies J.L., Manning B.D. (2011). MTOR links oncogenic signaling to tumor cell metabolism. J. Mol. Med..

[97] Shimobayashi M., Hall M.N. (2014). Making new contacts: The mTOR network in metabolism and signalling crosstalk. Nat. Rev. Mol. Cell Biol..

[98] Damián J.P., Bausero M., Bielli A. (2015). Acute stress, hypothalamic-hypophyseal-gonadal axis and testicular function-a review. Ann. Anim. Sci..

